# 1,25-Dihydroxyvitamin D3 increases testosterone-induced 17beta-estradiol secretion and reverses testosterone-reduced connexin 43 in rat granulosa cells

**DOI:** 10.1186/1477-7827-12-90

**Published:** 2014-09-20

**Authors:** Ching-Tien Lee, Jiz-Yuh Wang, Kuang-Yi Chou, Ming-I Hsu

**Affiliations:** Department of Nursing, Hsin Sheng College of Medical Care and Management, Taoyuan, Taiwan; Department of Neurology, School of Medicine, College of Medicine, Kaohsiung Medical University, Kaohsiung, Taiwan; General Education Center, National Taipei University of Nursing and Health Sciences, Taipei, Taiwan; Department of Obstetrics and Gynecology, Wan Fang Hospital, Taipei Medical University, Taipei, Taiwan

**Keywords:** 17beta-estradiol, Estrogenesis, Connexin 43, Testosterone, 1,25-dihydroxyvitamin D3, Polycystic ovary syndrome, Calcium, Granulosa cells

## Abstract

**Background:**

Aromatase converts testosterone into 17beta-estradiol in granulosa cells, and the converted 17beta-estradiol contributes to follicular maturation. Additionally, excessive testosterone inhibits aromatase activity, which can lead to concerns regarding polycystic ovary syndrome (PCOS). Generally, 1,25-dihydroxyvitamin D3 (1,25D3) supplements help to improve the symptoms of PCOS patients who exhibit low blood levels of 1,25D3. Therefore, this study investigated the interaction effects of 1,25D3 and testosterone on estrogenesis and intercellular connections in rat granulosa cells.

**Methods:**

Primary cultures of granulosa cells were treated with testosterone or testosterone plus 1,25D3, or pre-treated with a calcium channel blocker or calcium chelator. Cell lysates were subjected to western blot analysis to determine protein and phosphorylation levels, and 17beta-estradiol secretion was examined using a radioimmunoassay technique. Cell viability was evaluated by MTT reduction assay. Connexin 43 (Cx43) mRNA and protein expression levels were assessed by qRT-PCR, western blot, and immunocytochemistry.

**Results:**

Testosterone treatment (0.1 and 1 microg/mL) increased aromatase expression and 17beta-estradiol secretion, and the addition of 1,25D3 attenuated testosterone (1 microg/mL)-induced aromatase expression but improved testosterone-induced 17beta-estradiol secretion. Furthermore, testosterone-induced aromatase phosphotyrosine levels increased at 10 min, 30 min and 1 h, whereas 1,25D3 increased the longevity of the testosterone effect to 6 h and 24 h. Within 18–24 h of treatment, 1,25D3 markedly enhanced testosterone-induced 17beta-estradiol secretion. Additionally, pre-treatment with a calcium channel blocker nifedipine or an intracellular calcium chelator BAPTA-AM reduced 1,25D3 and testosterone-induced 17beta-estradiol secretion. Groups that underwent testosterone treatment exhibited significantly increased estradiol receptor beta expression levels, which were not affected by 1,25D3. Neither testosterone nor 1,25D3 altered 1,25D3 receptor expression. Finally, at high doses of testosterone, Cx43 protein expression was decreased in granulosa cells, and this effect was reversed by co-treatment with 1,25D3.

**Conclusions:**

These data suggest that 1,25D3 potentially increases testosterone-induced 17beta-estradiol secretion by regulating aromatase phosphotyrosine levels, and calcium increase is involved in both 1,25D3 and testosterone-induced 17beta-estradiol secretion. 1,25D3 reverses the inhibitory effect of testosterone on Cx43 expression in granulosa cells.

## Background

Biosynthesis of 17β-estradiol from androgen precursors is catalysed by the enzyme aromatase, which is expressed from the *cyp19* gene and exists in the ovaries, placenta, testes, breasts, brain, fat, liver and muscles [1]. 17β-Estradiol production and follicular development are controlled by the expression level and activity of aromatase [2]. During follicular growth, aromatase mRNA expression levels in granulosa cells from dominant follicles and 17β-estradiol levels in the follicular fluid are significantly increased [3, 4].

Various studies have demonstrated the role of androgens in stimulating follicular development. Androgen receptors (AR) are observed in primary follicles and advanced-stage follicles, and are detected in the granulosa cells of primordial follicles [5]. Androgens have been shown to stimulate the growth of small antral follicles and inhibit apoptosis of preovulatory follicles in primate ovaries [6, 7], whereas AR-knockout mice exhibit greatly increased apoptosis of granulosa cells in preovulatory follicles [8]. Testosterone and dihydrotestosterone (DHT) stimulate the growth of cultured follicles, increase the number of follicles and increase granulosa cell proliferation in mammalian cells [6, 7]. Testosterone and androstenedione significantly increase the abundance of aromatase mRNA and the accumulation of 17β-estradiol [2, 9, 10]. However, excessive testosterone is involved in polycystic ovary syndrome (PCOS), which is the most common endocrine disorder in females and is associated with arrested follicular development and the failure to select a dominant follicle [11]. Deficiency of 1,25-dihydroxyvitamin D_3_ (1,25D_3_), an active form of vitamin D, is a common risk factor in patients with PCOS [12, 13]. A daily 1,25D_3_ supplement enhances the intestinal absorption of calcium and alleviates both PCOS symptoms and gonadal dysfunction in 1,25D_3_ receptor (VDR)-null mutant mice [14].

1,25D_3_ plays important roles in calcium homeostasis, bone metabolism, and cell differentiation, proliferation, and apoptosis. Immunohistochemistry assays have demonstrated that VDR localizes to the follicles and predominantly exists in the nuclei of granulosa cells, suggesting that it has some role in reproduction [15]. Recent studies have demonstrated that aromatase activity and expression level are low in the ovaries of VDR-null mutant mice, but the activity is increased to 60% of wild-type levels by calcium supplementation in the normal range. Circulating 17β-estradiol levels are also low in VDR-null mutant mice and do not rise after calcium supplementation [16]. Taken together, these results indicate that 1,25D_3_ plays a role in estrogenesis that is only partially mediated by extracellular calcium homeostasis.

It has recently been shown that signalling via 1,25D_3_/VDR-mediated protein tyrosine phosphorylation occurs in bone, intestine, muscle and cancer cells [17]. The fast nongenomic responses of 1,25D_3_/VDR in muscle cells involve the phosphotyrosine form of a downstream protein, c-Src [18, 19]. Interestingly, c-Src is also downstream of the estradiol/membrane-associated estradiol receptor (ER) in the stimulation of aromatase activity. The phosphorylation of aromatase at tyrosine 361 is crucial in the up-regulation of aromatase activity through the estradiol/membrane-associated ER and c-Src signalling in mammalian cell lines [20]. The AR is also localized in the plasma membrane and is associated with Src after testosterone treatment in Sertoli cells [21].

A gap junction is a type of intercellular connection that enables the transfer of various small molecules and ions between cells. Such transfers between granulosa cells and oocytes have been implicated in playing important roles in follicular development and oocyte growth [22]. Gap junctions between granulosa cells contain abundant levels of connexin (Cx) 43, which is present at every stage of follicular growth [23]. The level of Cx43 protein expression increases during follicular development and decreases with follicular atresia [24]. Oocytes from Cx43-null mice failed to reach meiotic maturation, resulting in infertility [24]. Wu et al. demonstrated that excess DHT reduces Cx43 expression and impairs communication between granulosa cells [25]. Additionally, 1,25D_3_ increases Cx43 protein levels and Cx43 mRNA stability via the nuclear VDR in human skin fibroblasts [26].

However, no studies have investigated the effect of the testosterone and 1,25D_3_ interaction on aromatase expression and phosphorylation in granulosa cells. Additionally, the effects of 1,25D_3_ and testosterone-regulated 17β-estradiol production and intercellular communication in granulosa cells have not been fully elucidated. We therefore investigated the interactions of 1,25D_3_ and testosterone to clarify their effects on aromatase protein expression and tyrosine phosphorylation, 17β-estradiol secretion and Cx43 protein expression in cultured ovarian granulosa cells.

## Methods

### Animal preparation

Immature female Sprague–Dawley rats were housed in plastic cages and maintained on a 12 h light/12 h dark cycle (light on at 6:00 a.m.) with food and water available continuously. The experimental procedures were consistent with the Guidelines of Animal Use and Care from the National Institute of Health and were approved by the Institutional Animal Care and Use Committee of Taipei Medical University-Wang Fang Hospital, Taiwan. Immature female rats at 23–24 days of age were injected with 15 IU PMSG (Sigma, St. Louis, MO, USA) for 48 h to stimulate the development of preantral follicles to antral follicles, and the ovaries of the animals were removed after the animals were sacrificed.

### Granulosa cell culture

The ovaries were cleared from the surrounding fat and oviduct, and punctured with 27-gauge needles in phenol red-free Dulbecco's Modified Eagle's medium (DMEM)/F12 medium (Invitrogen, Carlsbad, CA, USA), modified as described previously [27]. Granulosa cells were pelleted by centrifugation at 1000 rpm (4°C, 10 min) and resuspended in phenol red-free DMEM/F12 medium with 0.1% fatty acid-free bovine serum albumin, 1% fetal bovine serum, and 2 μg/mL bovine insulin. The follicular debris was then removed, and the granulosa cell suspensions were filtered through a cell strainer (BD Falcon, Bedford, MA, USA). The cells were plated at a concentration of 2.5 × 10^6^ per well in a 6-well plate and were allowed to attach and grow to confluence for 1 day at 37°C, 5% CO_2_, and 95% air. The cultured cells were then incubated in serum/phenol red-free medium (DMEM/F12 containing 0.1% lactalbumin enzymatic hydrolysate) overnight before the beginning of treatment. The cells were treated with different doses of testosterone (0.01, 0.1 or 1 μg/mL) or testosterone combined with 1,25D_3_ (0.1 μM) in 2 mL of serum/phenol red-free medium for 24 h. Granulosa cells were pretreated with an L-type calcium channel blocker nifedipine (NIF, 10 μM, Sigma, St. Louis, MO, USA) or an intracellular calcium chelator bis-(a-aminophe-noxy)-ethane-N,N,N',N'-tetraacetic acid, tetra(acetoxymethyl)-ester (BAPTA-AM, 10 μM, Tocris, Minneapolis, MN, USA) for 30 min, pretreated with 1,25D_3_ for 15 min, and treated with one of three testosterone doses or vehicle for 24 h. At the end of the incubation period, the medium was collected, and the cells were lysed in ice-cold lysis buffer containing 50 mM Tris–HCl (pH 8.0), 150 mM NaCl, 0.1% SDS, 0.5% sodium deoxycholate, 0.1% Triton X-100, protease inhibitors and phosphatase inhibitors. A protein extract from the supernatant was used for western blot analysis.

### Western blot

The protein content of the extracts was determined using the Bio-Rad Protein Assay Reagent. Equal amounts of the protein extract (15 μg) were mixed with loading buffer and subjected to 10% sodium dodecyl sulfate-polyacrylamide gel electrophoresis, followed by transfer to a PVDF (polyvinylidene difluoride) membrane (Millipore, Billerica, MA, USA). After blocking for 1 h with 5% non-fat milk powder in Tris Buffered Saline (25 mM Tris, 135 mM NaCl and 2.5 mM KCl) with 0.05% Tween-20 (TBST), the membranes were incubated overnight with primary antibodies in TBST containing 5% non-fat milk powder and subsequently with horseradish peroxidase (HRP)-conjugated secondary antibody (Millipore, Billerica, MA, USA) in TBST with 5% non-fat milk powder at room temperature for 1 h. For experiments involving re-immunoblotting to different antibodies, the blots were stripped in 0.2 M glycine (pH 2.5) and 0.05% Tween-20 at 80°C for 20 min and then rinsed twice with 0.09 M boric acid (pH 7.4), 0.9% NaCl, and 0.05% Tween-20. The membranes were immunoblotted with different antibodies: aromatase (1:1000, Serotec, kidlington, Oxford, UK), phosphotyrosine (1:5000, Millipore, Billerica, MA, USA), Cx43 (1:1000, Novex, San Diego, CA, USA), estradiol receptor β (ERβ) (1:1000, GeneTex, San Antonio, Texas, USA), VDR (1:1000, GeneTex, San Antonio, Texas, USA), and β-actin (1:10000, Chemicon, Temecula, CA, USA). Immunoreactivity was detected by chemiluminescence autoradiography (ECL kit, Millipore, Billerica, MA, USA) in accordance with the manufacturer’s instructions. The protein bands were quantified using the NIH image J Software.

### Immunoprecipitation-western blot analysis

Granulosa cells were lysed in ice-cold lysis buffer containing 50 mM Tris–HCl (pH 8.0), 150 mM NaCl, 0.1% SDS, 0.5% sodium deoxycholate, 0.1% Triton X-100, protease inhibitors, and phosphatase inhibitors. The clarified lysate was immunoprecipitated at 4°C for 4 h in 300 μl of NP40 buffer (150 mM NaCl, 1% NP40, 50 mM Tris–HCl, pH 8.0, protease inhibitors, and phosphatase inhibitors) with anti-aromatase antibody (Serotec, kidlington, Oxford, UK) and protein G (GE Healthcare, Piscataway, NJ, USA). After washing, the immunoprecipitates were boiled, separated by 10% SDS-PAGE, and transferred to PVDF membranes. An Easyblot kit (GeneTex, San Antonio, Texas, USA) was used to decrease the interference caused by the heavy and light chains of the IgG. EasyBlocker was used as a blocking buffer to minimize the background caused by protein G contamination, and an EasyBlot anti-mouse IgG (HRP) was used that specifically reacts with the native form of mouse IgG and does not bind to the denatured form.

### Aromatase activity assay

The enzyme aromatase is responsible for the synthesis of estradiol from testosterone. Hence, the effect of 1,25D_3_ on 17β-estradiol secretion by granulosa cells was used as an indicator of aromatase activity (modified by Zaher Merhi et al., 2014) [28]. To study the effect of 1,25D_3_ on the aromatase activity, granulosa cells were cultured in 24-well culture plates for 24 h to attach to the plate. After 24 h of culture, the cells were treated with testosterone or 1,25D_3_/testosterone (i.e. 1,25D_3_ plus testosterone), the medium was collected at the indicated times (10 min, 30 min, 1 h, 6 h and 24 h), and the 17β-estradiol level was measured. In order to exclude the earlier difference in 17β-estradiol secretion, the culture medium was removed after 18 h and replaced with fresh medium supplemented with testosterone or 1,25D_3_/testosterone for another 6 h (18–24 h) treatment. The medium was collected for the measurement of 17β-estradiol concentrations at 18–24 h after the addition of the testosterone or 1,25D_3_/testosterone.

### Radioimmunoassay

The cell culture medium was collected and stored at −80°C until the assay was performed. 17β-Estradiol levels were assayed using a Coat-A-Count Estradiol RIA kit (Siemens, Dublin, Ireland) according to the manufacturer’s protocol. 1 mL of ^125^I-labeled estradiol and 100 μL samples were incubated in anti-estradiol antibody-coated tubes for 3 h at room temperature. After decanting the mixture and washing the tubes, the radioactivity levels of the tubes were counted in a gamma counter. The counts are inversely related to the amount of 17β-estradiol present in the sample. The intra assay coefficients of variation for assays ranged between 3% and 16%, with a mean of 10.2%. The percentage cross-reactivity of this antiserum was 17α-estradiol: not detectable; estriol: 0.32%; estrone-β-D-glucuronide: 1.8%. The limit and highest of detection of the assay was 0–3600 pg/mL.

### 3-(4,5-dimethylthianol-2-yl)-2,5-diphenyltetrazolium bromide (MTT) reduction assay

Cell viability was measured by a colorimetric MTT reduction assay performed as first described by Mosoman [29]. Each culture well was incubated in 0.5 mg/mL MTT (Sigma, St. Louis, MO, USA) culture medium followed by incubation for 4 h in 5% CO_2_ at 37°C. The culture medium was then aspirated, and cells were lysed with DMSO. Quantitation of MTT reduction was assayed by measuring the absorbance at 570 nm (against the 630 nm reference) using an ELISA reader (BioTek, Winooski, VT, USA).

### RNA extraction and quantitative reverse transcription polymerase chain reaction (qRT-PCR)

Total cellular RNA from granulosa cell cultures was isolated using a Total RNA Purification Kit (GeneMark, GMbiolab, Taichung, Taiwan) according to the manufacturer’s instructions. RNA concentrations and purity were determined using a NanoDrop ND2000 Spectrophotometer (Thermo Scientific, Wilmington, DE, USA). First-strand cDNA was synthesized from 300 ng of total RNA using SuperScript III Reverse Transcriptase (Invitrogen, Carlsbad, CA, USA). The cDNA were used as templates in subsequent qPCR by an ABI 7500 PCR Detection System (Applied Biosystem, Foster City, CA, USA). The primer sequences for Cx43 used for PCR amplifications were as follows: 5′-TTG TTT CTG TCA CCA GTA AC-3′ (antisense) and 5′-GAT GAG GAA GGA AGA GAA GC-3′ (sense). GAPDH was used as the internal control: 5′-CCG CCT GCT TCA CCA CCT TCT-3′ (antisense) and 5′-GTC ATC ATC TCC GCC CCT TCC-3′ (sense). Each reaction contained the RT mixture, primers and SYBR Green Master Mix (Invitrogen, Carlsbad, CA, USA) and was carried out with the following profile: initial heating to 95°C for 10 min followed by 40 cycles of heating to 95°C for 15 s, incubation at 55°C for 30 s and incubation at 72°C for 90 s. Melting-curve analysis and PCR products were run on agarose gels with ethidium bromide staining. Cx43 mRNA levels were normalized to GAPDH and expressed as values relative to the control using the comparative threshold cycle method.

### Immunocytochemistry

Granulosa cells were cultured on poly-L-lysine coated coverslips in 6-well plates. After 24 h of treatment, the granulosa cells were fixed in cold 4% paraformaldehyde for 20 min, rinsed three times with phosphate-buffered saline, and permeabilized with 0.1% Triton. Cells were blocked for 1 h with 5% goat serum and incubated for overnight at 4°C with rabbit anti-Cx43 (diluted 1:100, Novex, San Diego, CA, USA). The cells were then incubated for 1 h with FITC-conjugated goat anti-mouse IgG. Nuclei were stained with DAPI. Immunofluorescence images were captured using a confocal microscope (Zeiss LSM 700, Germany).

### Statistical analysis

All biochemical data were analyzed with Student’s t-test or a one-way analysis of variance (one-way ANOVA). Specific comparisons between any experimental group and a common control group were made using Dunnett’s *t*-test. Comparisons between two experimental groups were made using the Student-Newman-Keuls method. Statistical significance was evaluated at the levels of *p* < 0.05, *p* < 0.005, and *p* < 0.001.

## Results

### 1,25D_3_ attenuated testosterone-induced aromatase expression and increased 17β-estradiol secretion

**Figure 1:**
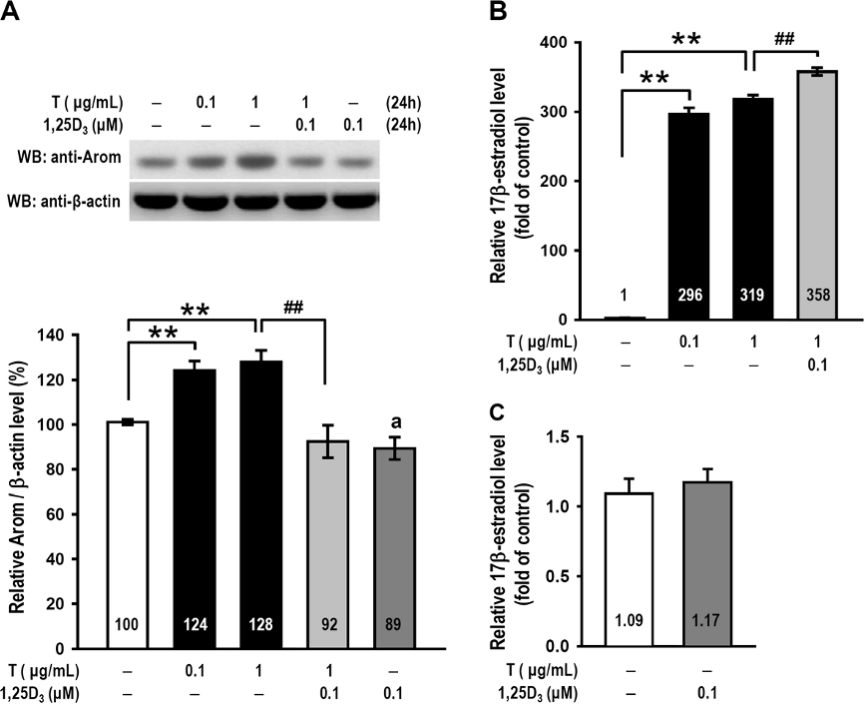
**1,25D**_**3**_ **attenuated testosterone-induced aromatase expression but improved 17β-estradiol secretions.** Ovarian granulosa cells from rats were pretreated with 1,25D_3_ (0.1 μM) for 15 min and subsequently treated with 0.1 μg/mL or 1 μg/mL testosterone (denoted by T) or vehicle (denoted by −) for 24 h. **(A)** Cells were lysed for protein extraction, and cell lysates were subjected to western blot analysis for aromatase protein detection. The level of aromatase (denoted by Arom) expression was estimated by densitometric analyses after normalization with the β-actin signal (n = 5–6 in each group). **(B and C)** 17β-Estradiol secretions from the granulosa cells were collected from the medium and assessed over the course of a 24-h period of drug treatments. 17β-Estradiol release data were expressed as the fold-change relative to the vehicle-treated control (n = 4–5 in each group). The values represent the means ± SEM. **, *p* < 0.005 for comparison with the vehicle-treated group; ##, *p* < 0.005 for comparison of testosterone/1,25D_3_ and testosterone alone; a, *p* < 0.05 for comparison of 1,25D_3_ alone and vehicle-treated group. The values listed within column indicate the means.

To examine whether 1,25D_3_ and testosterone regulate aromatase expression and 17β-estradiol secretion in granulosa cells, we treated cells with 0.1 or 1 μg/mL testosterone or 0.1 μM 1,25D_3_ plus 1 μg/mL testosterone for 24 h. Western blot analysis revealed that both doses of testosterone increased the aromatase levels (*p* < 0.005; Figure 1A) compared to vehicle-treated cells, and addition of 0.1 μM 1,25D_3_ resulted in a significant decrease of the 1 μg/mL testosterone-induced aromatase levels (*p* < 0.005; Figure 1A). Additionally, treatment with 1,25D_3_ alone attenuated aromatase expression compared to vehicle treatment (*p* < 0.05; Figure 1A). We also examined 17β-estradiol secretion using a radioimmunoassay and found that both testosterone-treated groups clearly experienced enhanced 17β-estradiol secretion (*p* < 0.005; Figure 1B). The 1,25D_3_/testosterone group exhibited significantly increased 17β-estradiol secretion relative to the group without 1,25D_3_ treatment (*p* < 0.005). Treatment of cells with 0.1 μM 1,25D_3_ alone for 24 h did not result in a significant increase in 17β-estradiol secretion (*p* > 0.05; Figure 1C). These results indicate that 1,25D_3_ has the potential to improve testosterone-induced 17β-estradiol secretion but causes a decrease in testosterone-induced aromatase expression.

### 1,25D_3_ significantly increased aromatase tyrosine phosphorylation

**Figure 2:**
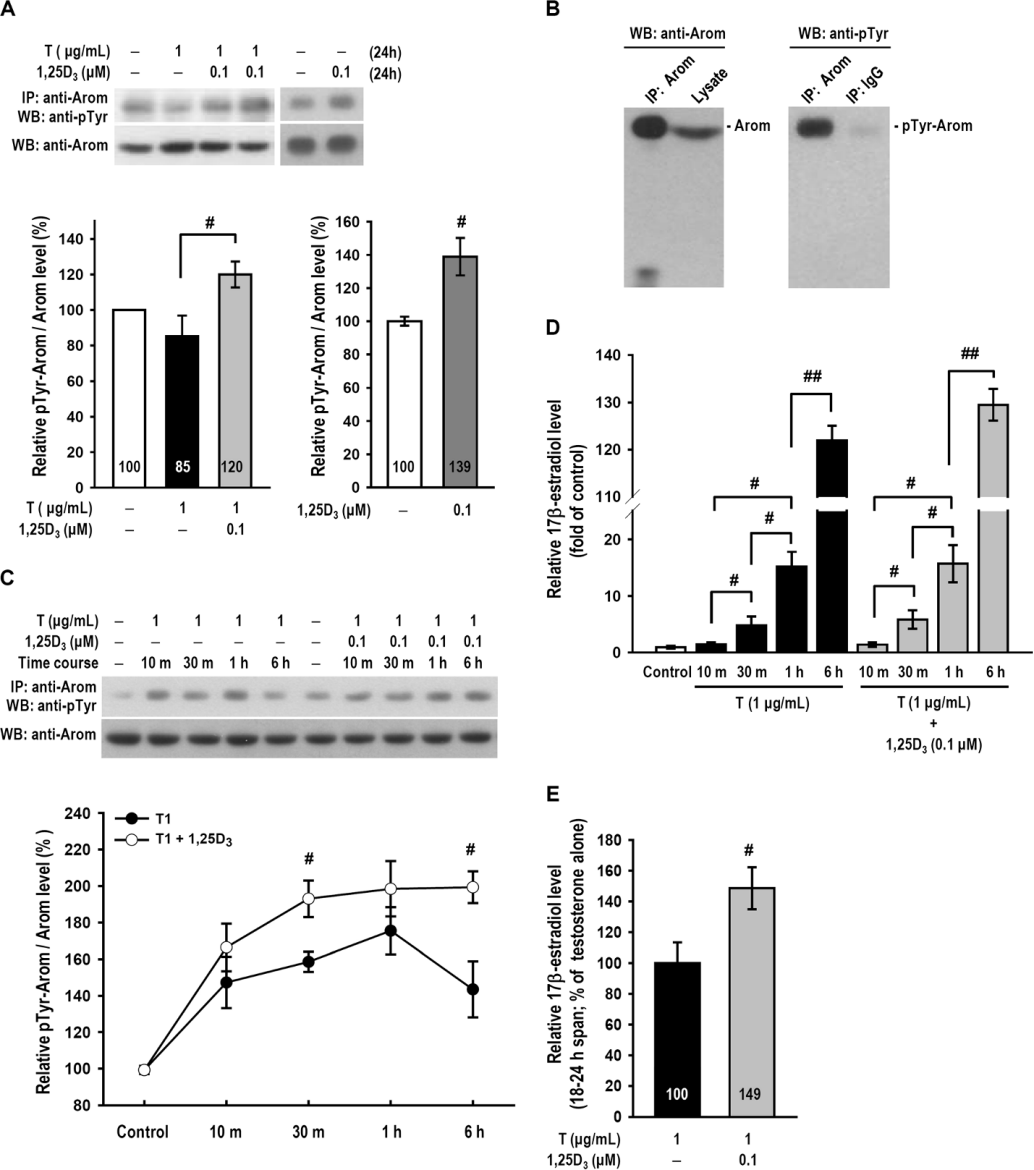
**1,25D**_**3**_ **enhanced testosterone-induced aromatase tyrosine phosphorylation and 17β-estradiol secretion during 18–24 h. (A)** Rat ovarian granulosa cells were treated with vehicle, testosterone, testosterone plus 1,25D_3_ or 1,25D_3_ alone for 24 h (n = 4–5 in each group). Aromatase was immunoprecipitated before being subjected to SDS-PAGE for resolution. The resultant blot was probed using both phosphotyrosine and aromatase antibodies. The expression of aromatase tyrosine phosphorylation (denoted by pTyr-Arom) was normalized to the corresponding aromatase expression and is represented as a percentage relative to the vehicle-treated control. **(B)** The left panel is a positive control that was both immunoprecipitated and immunoblotted with an anti-aromatase antibody. The lysate was also immunoprecipitated with the anti-aromatase antibody or IgG and immunoblotted with an anti-phosphotyrosine antibody, as shown in the right panel. A weak band in the IgG lane is as a negative control. **(C)** Cells were treated with testosterone alone or testosterone plus 1,25D_3_ as indicated for various time periods (n = 4–5 in each group). Each pTyr-Arom level was normalized using the corresponding aromatase level and is represented as a percentage relative to the vehicle-treated control. **(D)** After treatment for various time periods as indicated, the 17β-estradiol production levels were measured by radioimmunoassay. **(E)** After treatment with testosterone alone or testosterone plus 1,25D_3_ for 18 h, the culture medium was removed and then replaced with fresh medium. Cells were continuously treated for another 6 h (18–24 h span). After the final 6 h, medium was collected and the 17β-estradiol secretion levels were measured by radioimmunoassay (n = 6 in each group). The values represent the means ± SEM. #, *p* < 0.05; ##, *p* < 0.005 for comparison of the two treatment groups. The values listed within column indicate the means.

We evaluated whether the phosphorylation of aromatase was regulated by testosterone and 1,25D_3_ using immunoprecipitation-western blot analysis. We determined that the 1,25D_3_/testosterone group exhibited significantly increased the level of aromatase tyrosine phosphorylation at 24 h relative to the testosterone group (*p* < 0.05; Figure 2A, left), and 1,25D_3_ alone also significantly increased the level of aromatase tyrosine phosphorylation relative to the control group (*p* < 0.05; Figure 2A, right). The left panel in Figure 2B is a positive control that was both immunoprecipitated and immunoblotted with an anti-aromatase antibody, and the right panel is a negative control for IgG. The lysate of the granulosa cells treated with 1,25D_3_/testosterone showed a significant band attributed to immunoprecipitated aromatase phosphotyrosine and a weak band for IgG.

We next investigated the time course of the phosphotyrosine response to testosterone and to 1,25D_3_/testosterone. Our data demonstrate that testosterone treatment induced aromatase tyrosine phosphorylation at 10 min, 30 min and 1 h, whereas 1,25D_3_ improved the effect of testosterone at 30 min and increased the duration of the effect to 6 h (*p* < 0.05; Figure 2C) and 24 h (Figure 2A). We also collected the medium for the 17β-estradiol assay and determined that testosterone-induced 17β-estradiol secretion increased at 30 min, 1 h (*p* < 0.05) and 6 h (n = 5; *p* < 0.001; Figure 2D). There was no difference in 17β-estradiol secretion between the testosterone-treated groups and the 1,25D_3_/testosterone-treated groups at up to 6 h (*p* > 0.05; Figure 2D). We also collected the medium 18–24 h after drug treatment and found that the 1,25D_3_/testosterone-treated group significantly increased 17β-estradiol secretion (*p* < 0.05; Figure 2E). These studies demonstrated that 1,25D_3_ increased aromatase tyrosine phosphorylation and 17β-estradiol secretion.

### 1,25D_3_ and testosterone-induced 17β-estradiol production was mediated by calcium

**Figure 3:**
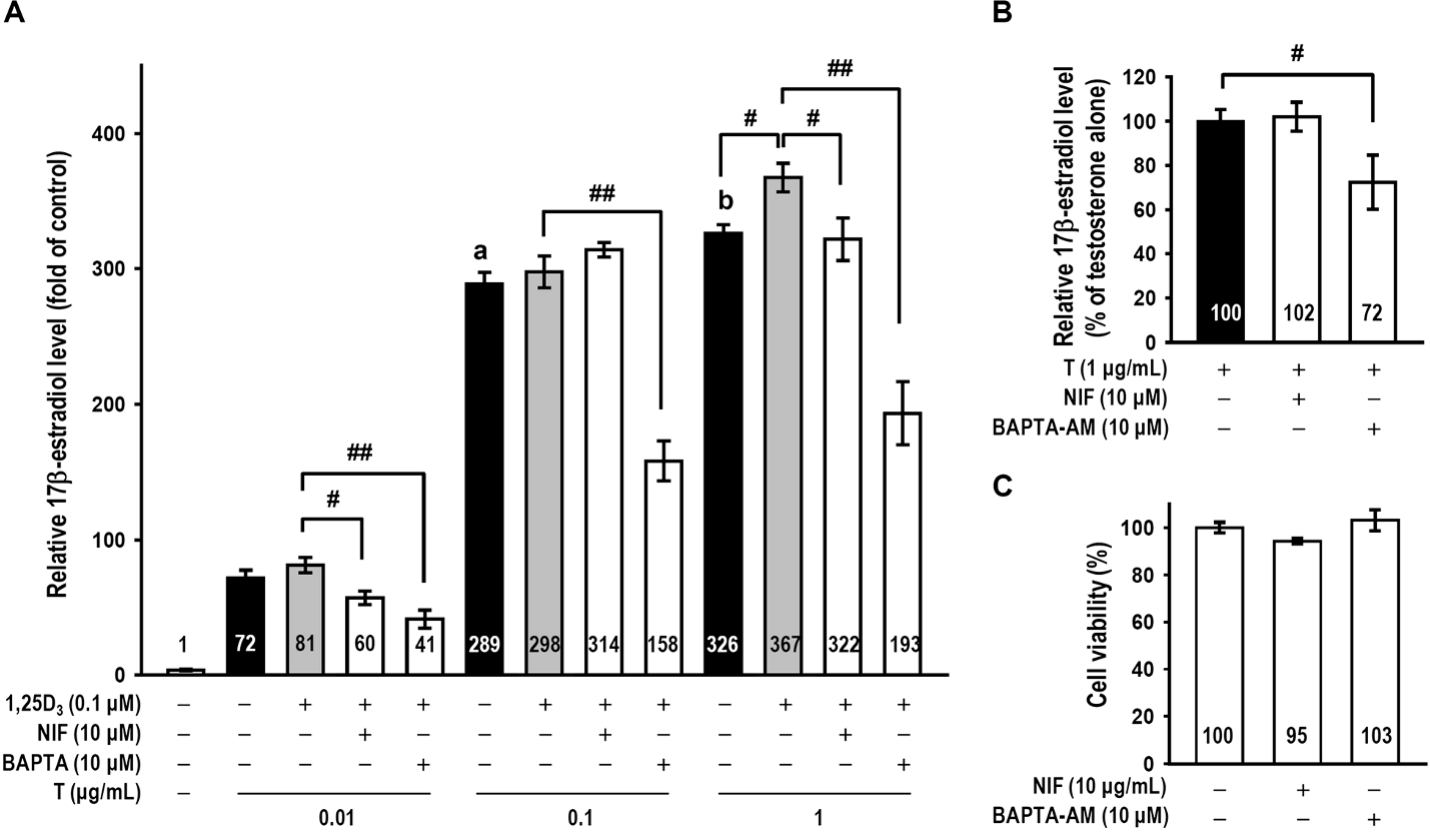
**1,25D**_**3**_ **and testosterone-induced 17β-estradiol secretion was mediated by calcium accumulation. (A)** Granulosa cells were divided into vehicle, testosterone, testosterone plus 1,25D_3_, L-type calcium channel blocker NIF (10 μM) or intracellular calcium chelator BAPTA-AM (10 μM) pre-treatment groups (n = 4–5 in each group). The individual levels of 17β-estradiol were also assayed using a radioimmunoassay. **(B)** Testosterone-induced 17β-estradiol released by granulosa cells cultured with or without NIF or BAPTA-AM in the absence of 1,25D_3_ was measured (n = 9 in each group). **(C)** After NIF or BAPTA-AM treatment for 24 h, cells were subjected to the MTT reduction assay to measure cell viability. MTT reduction data are expressed as the percentage of values relative to the control group. The measurements were performed in triplicate, and each cytotoxicity experiment was repeated four times. The values represent the means ± SEM. #, *p* < 0.05; ##, *p* < 0.005 for comparison of two drug groups; a, *p* < 0.001 for comparison of 0.1 μg/mL and 0.01 μg/mL testosterone; b, *p* < 0.05 for comparison of 1 μg/mL and 0.1 μg/mL testosterone. The values listed within column indicate the means.

One possible mechanism for 1,25D_3_/testosterone modulation of 17β-estradiol production is through increasing calcium concentrations. We studied whether NIF or BAPTA-AM could inhibit 1,25D_3_/testosterone-regulated 17β-estradiol secretion. A radioimmunoassay of 17β-estradiol production is presented in Figure 3. Statistical analysis revealed that testosterone doses (0.01, 0.1 and 1 μg/mL) increased 17β-estradiol production (*p* < 0.001 and *p* < 0.005), and 1,25D_3_ (0.1 μM) increased high-dose (1 μg/mL) testosterone-induced 17β-estradiol secretion (*p* < 0.05; Figure 3A). Our data demonstrate that pre-treatment with NIF reduced 1,25D_3_ and testosterone (0.01 and 1 μg/mL)-enhanced 17β-estradiol secretion in granulosa cells (*p* < 0.05; Figure 3A) but not testosterone (1 μg/mL)-induced 17β-estradiol secretion (Figure 3B). BAPTA-AM reduced 17β-estradiol secretion induced by 1,25D_3_/testosterone (0.01, 0.1 and 1 μg/mL) (*p* < 0.001; Figure 3A) and by testosterone (1 μg/mL) alone (*p* < 0.05; Figure 3B). We also monitored cell viability using an MTT reduction assay. The viability of cultured cells exposed to NIF (10 μM) or BAPTA-AM (10 μM) did not significant differ from the control group (*p* > 0.05; Figure 3C). Thus, these data revealed that chelation of intercellular calcium with BAPTA-AM resulted in suppression of both testosterone- and 1,25D_3_/testosterone-modulated 17β-estradiol secretion, while calcium from the L-type calcium channel was involved in only 1,25D_3_/testosterone-modulated 17β-estradiol secretion in cultured granulosa cells.

### The effects of testosterone and 1,25D_3_ on ERβ or VDR expression

**Figure 4:**
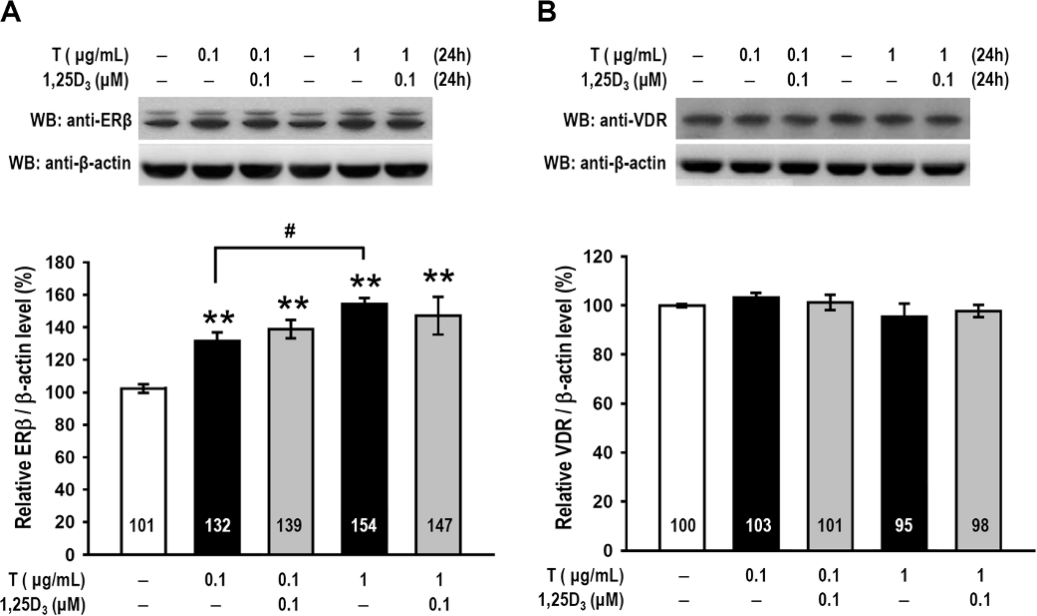
**Testosterone, but not 1,25D**_**3**_ **, enhanced ERβ expression; testosterone and 1,25D**_**3**_ **had no effect on VDR expression.** Granulosa cells were pretreated with 1,25D_3_ for 15 min and subsequently treated with one of two testosterone doses or vehicle for 24 h (n = 4–5 in each group). The granulosa cell lysates were subjected to western blot analysis for **(A)** ERβ and **(B)** VDR expression levels. The levels of ERβ and VDR expression were quantified using densitometry and normalized to β-actin. The values represent the means ± SEM. **, *p* < 0.005 for comparison with the vehicle-treated group. #, *p* < 0.05 for comparison of two drug groups. The values listed within column indicate the means.

We examined the expression levels of ERβ and VDR in granulosa cells to determine if they were altered by testosterone and 1,25D_3_. Western blot analysis revealed that granulosa cells treated with testosterone (0.1 and 1 μg/mL) exhibited markedly increased ERβ expression levels (*p* < 0.005; Figure 4A) and that 1 μg/mL testosterone treatment increased ERβ expression more than treatment with 0.1 μg/mL testosterone (*p* < 0.05). However, the two doses of testosterone did not alter VDR levels (*p* > 0.05; Figure 4B). Additionally, pre-treatment with 1,25D_3_ did not alter testosterone-induced ERβ expression and did not influence VDR expression (n = 3–4, *p* > 0.05; Figure 4A, B). Thus, testosterone alone affected ERβ expression, but 1,25D_3_ pre-treatment had no influence on ERβ or VDR in cultured granulosa cells.

### 1,25D_3_ reversed testosterone-induced down-regulation of Cx43 in granulosa cells

**Figure 5:**
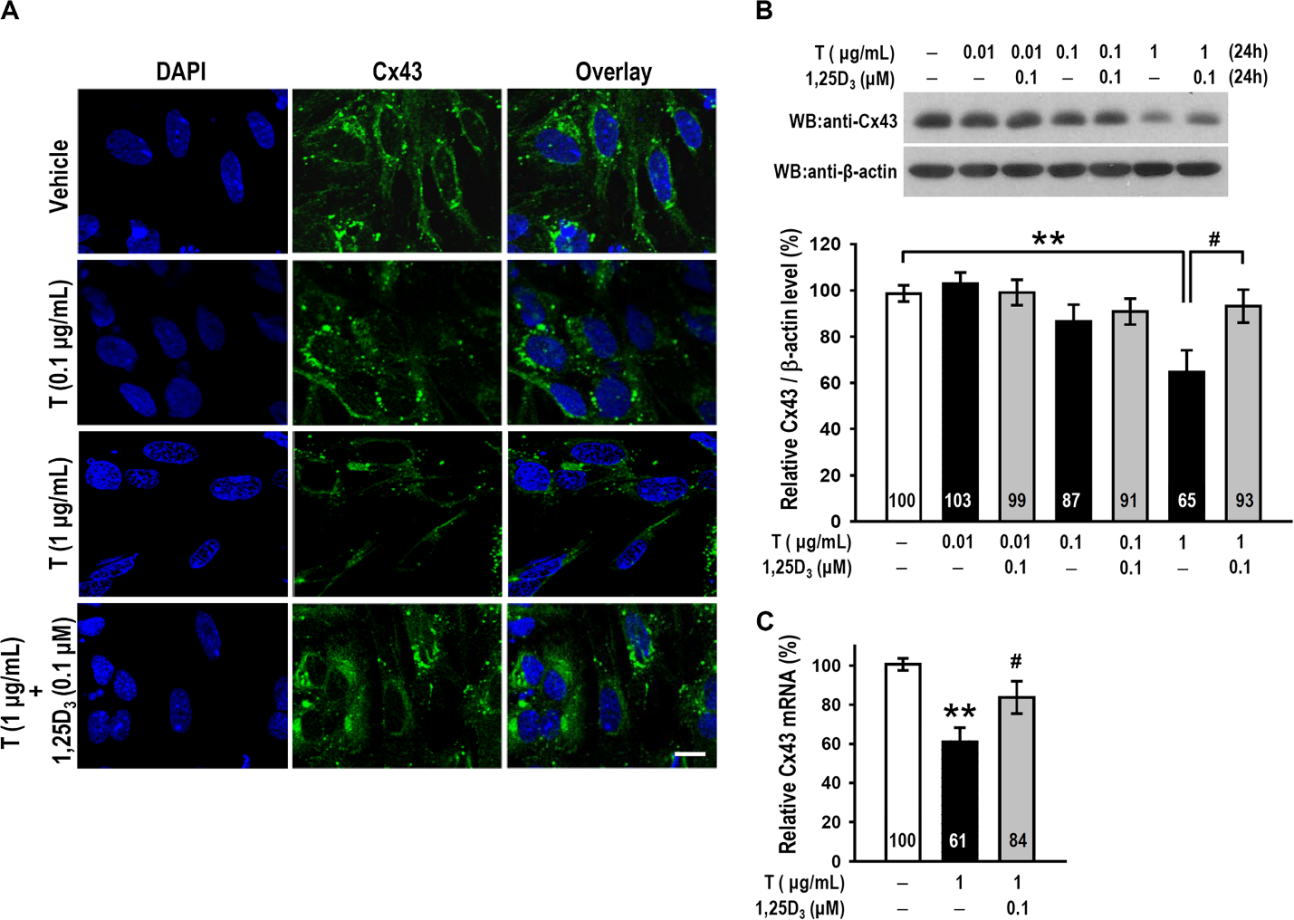
**Testosterone-induced down-regulation of Cx43 in granulosa cells was reversed by treatment with 1,25D**_**3**_ **. (A)** Granulosa cells were treated for 24 h with vehicle, testosterone (0.1 or 1 μg/mL) or testosterone (1 μg/mL) plus 1,25D_3_ (0.1 μM) and fixed in cold paraformaldehyde solution. Cell nuclei were stained with DAPI (left column), and Cx43 levels (green) were examined by confocal microscopy (middle column). The overlay is shown in the right column. **(B, C)** Granulosa cells were treated with 0.01, 0.1, or 1 μg/mL testosterone for 24 h in the presence of vehicle control or 0.1 μM 1,25D_3_. The levels of Cx43 protein and Cx43 mRNA were examined by western blot and qRT-PCR analysis, respectively. The results are expressed as the means ± SEM (n = 4–6 in each group). **, *p* < 0.005 for comparison with the vehicle-treated group. #, *p* < 0.05 for comparison of two drug groups. The values listed within column indicate the means. Scale bars, 10 μm.

Finally, to examine whether 1,25D_3_ can affect the testosterone-induced down-regulation of Cx43, granulosa cells were treated with testosterone in the presence or absence of 0.1 μM 1,25D_3_. We determined the cellular localization of Cx43 using immunofluorescence and observed its expression as green fluorescent plaques (Figure 5A). Cx43 protein expression was markedly decreased when cells were treated with a high dose of testosterone (1 μg/mL; *p* < 0.005), but this effect was reversed by pre-treatment with 1,25D_3_ (*p* < 0.05; Figure 5A, B). We performed a real-time PCR analysis and found that cultured cells pre-treated with 1,25D_3_ also reversed testosterone (1 μg/mL)-induced down-regulation of Cx43 (*p* < 0.05; Figure 5C). These results indicated that 1,25D_3_ abolishes the inhibitory effect of testosterone on Cx43 expression in granulosa cells.

## Discussion

Testosterone can induce concentration-dependent physiological and pathological effects. Physiologic levels of testosterone are converted into sufficient 17β-estradiol to improve follicular development, and the pathological effects of testosterone may be due to induced hyperandrogenism and arrested follicular development. Androgen concentrations in the follicular fluid (FF) are higher in small follicles than in large follicles, and the androgen concentration in PCOS FF is higher than in FF from healthy women [30, 31]. In the present study, we have demonstrated the dose dependence of testosterone-induced aromatase expression (Figure 1A) and 17β-estradiol secretion (Figures 1B and 3A). These results are consistent with a previous report demonstrating that testosterone increases aromatase mRNA levels [2]. Testosterone increases the levels of aromatase mRNA promoter 1.1- and 1.5-derived transcripts at 1 μM but only increases the promoter 2-derived transcript at the highest (100 μM) dose [9]. Interestingly, we found that 1,25D_3_ reduced testosterone-induced aromatase expression but stimulated 17β-estradiol secretion (Figure 1A, B). 1,25D_3_ appears to modulate aromatase expression in the estrogenesis of granulosa cells and has been shown to exert tissue-specific effects on aromatase expression by various promoters [32]. For example, 1,25D_3_ increases aromatase expression in placental cells and osteoblasts but down-regulates aromatase expression in breast cancer cells [33–35]. Lundqvist et al. also demonstrated that a 1,25D_3_ analogue reduces aromatase expression by promoting dissociation of the co-modulator Williams syndrome transcription factor from the *cyp19a1* promoter in breast cancer cells [36]. In addition, competition for shared co-regulators between VDR and AR is one possible explanation for the suppressive effect of 1,25D_3_/VDR signals on AR transcriptional activity [37]. In our study, we found that treatment with 1,25D_3_ alone reduced aromatase expression but did not alter 17β-estradiol secretion (Figure 1A, C). Thus, 1,25D_3_ exhibits a synergist effect in attenuating testosterone-induced aromatase expression.

Recently, aromatase activity was shown to mediate posttranslational modifications. Changes in aromatase activity often reflect differential protein expression arising from a slow rate of mRNA transcription. The phosphorylation of aromatase rapidly regulates estradiol production. For example, studies of murine aromatase suggest that serine 118 [38] or tyrosine 361 [20] can be phosphorylated and affect aromatase stability or activity. Specifically, increases in aromatase activity and estradiol secretion are regulated by c-Src kinase-catalysed tyrosine phosphorylation [20] and inhibits in aromatase activity by protein tyrosine phosphatase 1B in breast cancer cells [39]. In our study, although 1,25D_3_ inhibited aromatase protein expression, we also found that it can regulate tyrosine phosphorylation and change the activity of aromatase to improve 17β-estradiol secretion at longer times. We demonstrated that 1,25D_3_ treatment led to a significantly increased aromatase tyrosine phosphorylation level at 30 min, 6 h (Figure 2C) and 24 h (Figure 2A, left) compared with testosterone alone (Figure 2A, C). 1,25D_3_ also increased the level of aromatase tyrosine phosphorylation without testosterone at 24 h (Figure 2A, right). No difference in 17β-estradiol concentration between the testosterone and 1,25D_3_/testosterone groups was observed within the first 6 h (Figure 2D), but 1,25D_3_ markedly increased 17β-estradiol secretion at 18–24 h (Figure 2E). These results might suggest that a sustained, 1,25D_3_-induced increase in aromatase tyrosine phosphorylation maintains the effect of testosterone on 17β-estradiol secretion at 18–24 h. Thus, the 13% increase in 17β-estradiol production might arise at later times during the 24 h treatment with 1,25D_3_. Because VDRs are located in the largest follicles, the 13% increase in 17β-estradiol from 1,25D_3_/testosterone treatment might assist growth of the largest follicles. 1,25D_3_ might in this way inhibit aromatase expression and prevent aromatase excess syndrome, and increased aromatase tyrosine phosphorylation may rapidly regulate 17β-estradiol in the appropriate time frame.

1,25D_3_ is also known to promote calcium absorption from intestinal cells [40]; however, there was no clear evidence suggesting that 1,25D_3_ increased calcium accumulation in granulose cells. Two different calcium channels (T-type and L-type) are involved in steroidogenesis in granulose cells [41]. In this study, we observed that an L-type calcium channel blocker reduced 17β-estradiol secretion under 1,25D_3_/testosterone (0.01 or 1 μg/mL) treatment (Figure 3A). 1,25D_3_/testosterone-induced 17β-estradiol secretion was also significantly reduced by an intracellular calcium chelator. Additionally, NIF (10 μM) or BAPTA-AM (10 μM) alone did not cause cell toxicity (Figure 3C), and this dose of BAPTA-AM also reduces testosterone-induced 17β-estradiol secretion without 1,25D_3_ (Figure 3B). These results are similar to a report that implicated a calcium-dependent pathway in mediation of gonadotropin-induced steroidogenesis in the ovary [42]. Weitzel et al. demonstrated that calcium signals are critical in the inhibition of low-density lipoprotein receptor-1-mediated estradiol production in murine granulosa cells [43]. Our data is the first to illustrate that stimulation of 17β-estradiol production by 1,25D_3_ and testosterone is mediated by the L-type calcium channel and intracellular calcium levels (Figure 3).

Several effects of 17β-estradiol have been shown to be important for follicular development and ovarian function, including the regulation of ER levels, stimulation of DNA synthesis, cell proliferation and regulation of atresia in ovarian follicles [44]. ERβ is the important ER member expressed in growing granulosa cells and the mature follicle in rodent ovaries and is critical to granulosa cell proliferation and differentiation [45]. We have demonstrated that testosterone-treated granulosa cells exhibited significantly increased ERβ levels and that 1,25D_3_ did not alter testosterone-induced ERβ expression (Figure 4A). This result is similar to the finding that the expression levels of ERβ mRNA in the ovaries of VDR-null mutant and wild-type mice are the same [16]. Additionally, both testosterone and 1,25D_3_ had no effect on VDR expression (Figure 4B). These results might suggest that testosterone or 1,25D_3_/testosterone increases ERβ levels but not VDR levels.

Intercellular and intracellular endocrine regulatory mechanisms may be critical for follicle growth, dominant follicle selection, and follicle atresia. As ovarian follicles grow from the small to the large antral stage, granulosa cells provide 17β-estradiol by aromatase activation for dominant follicle development [46]. In contrast, other small follicles undergo atresia via apoptosis [47]. AR is expressed in primordial follicles, advanced-stage follicles and primary follicles [5], and testosterone stimulates the early stages of follicle growth, inhibits preovulatory follicle apoptosis and limits follicle size [6]. Thus, the observation that many small follicles grow in PCOS may be explained by excess testosterone. Alternatively, 1,25D_3_ might inhibit the testosterone-stimulated early stages of follicular growth because 1,25D_3_ is known to inhibit the proliferation and induce the differentiation of a variety of cells [48], and VDR is expressed in the granulosa cells of largest follicle [49]. It is possible that 1,25D_3_ paired with testosterone preferentially improves the growth of larger follicles. Hence, the effect of 1,25D_3_ and testosterone on aromatase in granulosa cells might indicate a plausible treatment option for PCOS. Several reports have also shown that 1,25D_3_ regulates antimüllerian hormone (AMH) signalling, follicle-stimulating hormone sensitivity, and progesterone production in human granulosa cells, and decreases the abnormally elevated AMH levels in 1,25D_3_-deficient women with PCOS, indicating a critical role for 1,25D_3_ in follicular development [50].

Intercellular communication via gap junctions is vitally important for granulosa cell differentiation and oocyte growth [22]. In this study, western blot analysis, qRT-PCR and immunostaining showed that high dose (1 μg/mL) of testosterone decreased Cx43 protein expression in granulosa cells, and this decrease was reversed by co-treatment with 0.1 μM 1,25D_3_ (Figure 5). These results are similar to the finding that excess DHT down-regulates Cx43 in granulosa cells [25]. The correlation between abnormal androgen concentrations and Cx43 expression might contribute to the pathogenesis of PCOS, and 1,25D_3_ might prevent Cx43 down-regulation.

## Conclusions

We determined that testosterone treatment increased aromatase expression and increased 17β-estradiol secretion in granulosa cells. 1,25D_3_ decreased testosterone-induced aromatase expression but significantly increased aromatase phosphotyrosine levels and lengthened the duration of testosterone-induced 17β-estradiol secretion in rat granulosa cells to 18–24 h. We also determined that 1,25D_3_/testosterone-induced 17β-estradiol secretion is dependent on changes in calcium levels. Additionally, testosterone increased ERβ expression, but neither 1,25D_3_ nor testosterone mediated VDR expression. Finally, a high dose of testosterone reduced Cx43 expression, and 1,25D_3_ abolished testosterone-reduced Cx43 levels.

## Abbreviations

AMH: Antimüllerian hormone
AR: Androgen receptors
BAPTA-AM: bis-(a-aminophe-noxy)-ethane-N,N,N',N'-tetraacetic acid tetra(acetoxymethyl)-ester
Cx43: Connexin 43
1: 25D_3_: 1,25-dihydroxyvitamin D_3_
DHT: Dihydrotestosterone
ERβ: Estradiol receptor β
FF: Follicular fluid
FSH: Follicle-stimulating hormone
NIF: Nifedipine
PCOS: Polycystic ovary syndrome
VDR: 1,25-dihydroxyvitamin D_3_ receptor.
